# Recent Advances in Mass Spectrometry-Based Structural Elucidation Techniques

**DOI:** 10.3390/molecules27196466

**Published:** 2022-09-30

**Authors:** Xin Ma

**Affiliations:** School of Chemistry and Biochemistry, Georgia Institute of Technology, 901 Atlantic Dr NW, Atlanta, GA 30332, USA; xin.ma@chemistry.gatech.edu; Tel.: +1-765-337-2895

**Keywords:** tandem mass spectrometry, structural elucidation, gas-phase ion chemistry, molecular characterization, omics

## Abstract

Mass spectrometry (MS) has become the central technique that is extensively used for the analysis of molecular structures of unknown compounds in the gas phase. It manipulates the molecules by converting them into ions using various ionization sources. With high-resolution MS, accurate molecular weights (MW) of the intact molecular ions can be measured so that they can be assigned a molecular formula with high confidence. Furthermore, the application of tandem MS has enabled detailed structural characterization by breaking the intact molecular ions and protonated or deprotonated molecules into key fragment ions. This approach is not only used for the structural elucidation of small molecules (MW < 2000 Da), but also crucial biopolymers such as proteins and polypeptides; therefore, MS has been extensively used in multiomics studies for revealing the structures and functions of important biomolecules and their interactions with each other. The high sensitivity of MS has enabled the analysis of low-level analytes in complex matrices. It is also a versatile technique that can be coupled with separation techniques, including chromatography and ion mobility, and many other analytical instruments such as NMR. In this review, we aim to focus on the technical advances of MS-based structural elucidation methods over the past five years, and provide an overview of their applications in complex mixture analysis. We hope this review can be of interest for a wide range of audiences who may not have extensive experience in MS-based techniques.

## 1. Introduction to Mass Spectrometry and Overview

Mass spectrometry (MS) has proven its unique capabilities in terms of analyzing complex mixtures with high accuracy and sensitivity, and it has been widely used in different studies. MS is such a versatile technique, and it manipulates ions (positively or negatively charged) in the gas phase by applying an external electric field or magnetic field. A wide range of compounds can be analyzed by MS with the proper application of ionization techniques (see Section 2.2 and Section 2.3). The ionization step can either take place under high vacuum or ambient conditions. More specifically, with the demands for the development of more efficient analytical methods for the study of biomolecules in their native environments, ambient ionization techniques have expanded drastically in the past decade [1]. After ionization, ions are accelerated, focused, and transferred to the mass analyzer sequentially. Ions with different *mass-to-charge* (*m/z*) ratios are then separated in the mass analyzer based on their different kinetic energies, momentums, flying times, or frequencies of a specific motion caused by the electric or magnetic field [2]. A mass spectrum is generated after the ions reach the detector, with the x-axis being the *m/z* of the ions, and the y-axis being the relative abundances (the abundances of the most abundant ions in the mass spectrum are assigned to 100% and referred to as the “base peak”) of the ions. One of the most highlighted features of MS is that it is capable of providing extensive structural information of the analyte ions. In addition to obtaining molecular weight (MW) from the molecular ions or the (de)protonated ions, the intact ions can also be monitored to undergo fragmentation processes so that key fragment ions can be produced, detected, and used to reconstruct the “unknown” molecule; therefore, MS has become a pivotal tool for the structural analysis of different compounds. A combination of several different mass analyzers (hybrid instruments) has enabled more accurate mass and structural analysis [3]. Several mass spectrometers also serve as chambers for performing gas-phase reactions so that additional structural information can be obtained when fragment ions of the analyte are not informative enough to elucidate the precursor’s structure (see Section 2.4 and Section 2.5, and examples in Section 3) [4,5,6]. Some highly reactive intermediates, that are not easy to capture in the condensed phase, can also be studied in the gas phase using MS, thus providing more insight into understanding the behavior of these “uncommon” species and how they may participate in critical chemical and biological processes [7,8,9,10,11]. By coupling modern separation techniques with MS, more powerful tools for the analysis of complex mixtures become approachable, thus enabling the studies of biomolecules in the presence of complex biomatrices (see Section 2.1). With the further improvement in mass resolving power and sensitivity, an increasing number of low-level biomolecules that may exhibit important biofunctions can now be unambiguously studied. In this article, we aim to provide an overview of structural elucidation capabilities for unknown molecules in complex mixtures by using MS-based techniques, and we review the technical advancements of MS and its recent applications in many different fields over the past five years, including the analysis of biomolecules, hydrocarbon molecules, and some natural products.

## 2. Technical Advances in MS-Based Structural Elucidation Techniques

### 2.1. Separation Techniques for MS-Based Mixture Analysis

Prior to MS analysis, a separation procedure is normally required for studying mixtures. The most common separation techniques that are widely coupled with MS include gas chromatography (GC), high performance liquid chromatography (HPLC), and capillary electrophoresis (CE). Among these so-called hyphenated methods, GC-MS is most commonly used for the separation and analysis of mixtures containing volatile and thermally stable components, such as petroleum samples and volatile organic compounds; therefore, although it is an inexpensive and very sensitive method, its main limitation stems from the fact that it has lower compound coverage compared with the other two techniques [12]. With the development of GC-MS methods targeted at nonvolatile analytes, the compound coverage issue has been largely resolved [13,14,15]. GC-MS is also limited in terms of its ability to analyze molecules that have relatively low MW, typically ≤1000 Da. HPLC-MS and CE-MS are free from the influence of sample volatility and MW, and thus, they have been more extensively used for current mixture analysis, especially HPLC-MS [16]. The sample preparation requirements for HPLC-MS are relatively easy; therefore, although it is a method that costs a considerably large amount of organic solvents, it remains the most important separation technique for mixture analysis. Other separation techniques, such as supercritical fluid chromatography (SFC), have also been used for MS-based mixture analysis [17]. Furthermore, with the rapid development of the ion mobility (IM) technique, an additional dimension of gas-phase separation, based on the molecular shapes of the analytes, has been incorporated into MS structural elucidation studies. The basic principles of IM techniques are briefly discussed in Section 2.6. A more detailed overview of IM techniques, applications, and future directions have been published [18]. We will showcase the applications of all mentioned hyphenated techniques in different research areas in Section 3.

### 2.2. EI Mass Spectrometry

As mentioned, mass spectrometry has become an essential tool for complex mixture analysis and structural elucidation. Gas-phase molecular ions (precursor ions), generated from the ion source of a mass spectrometer, can either remain intact or undergo fragmentation reactions depending on the ionization energy deposited to the neutral molecules. Characteristic fragment ions (product ions) generated in the ionization process are remarkable, as they normally provide important structural information relating to the precursor ions. More specifically, “hard” ionization techniques, such as electron (impact) ionization (EI), usually generates extensive fragment ions upon ionization, and sometimes the precursor ions (e.g., radical cations, M^+●^, in positive ion mode) are not even visible in the mass spectrum [19,20]. Moreover, because of its extremely high reproducibility, EI mass spectra have been used to establish libraries so that the identities of analyte ions can be, in many cases, confirmed by matching their measured EI spectra with the library data (false hits are still possible) [21]. In a typical (+)EI-MS experiment, a beam of high-energy electrons is generated from an electron filament (tungsten or rhenium), the gas-phase analyte molecules are bombarded by the high-energy electron beam as they pass through, and an electron is knocked off from the neutral analyte molecule. The energy of the electron beam used in EI can be as high as 70 eV, and almost all the organic molecules can be ionized efficiently and may be fragmented under such conditions (the average ionization energy of organic molecules is about 10 eV) [20].

**Figure 1:**
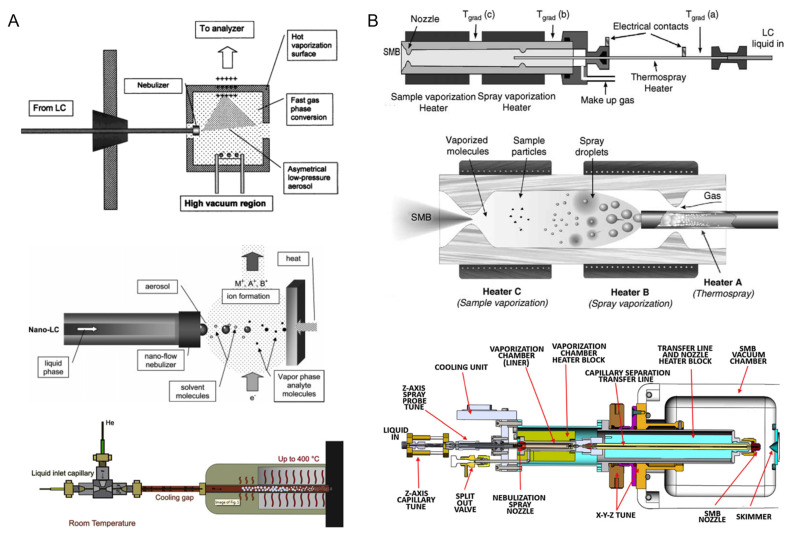
(**A**) Schematic of several versions of a direct EI interface for LC-EI-MS. (**B**) Schematic of two versions of supersonic molecular beam LC-EI-MS interfaces [27]. Adjusted and reprinted with permission from Elsevier.

EI has been successfully coupled to GC and used extensively for the analysis and molecular characterization of complex mixtures as a sensitive and inexpensive technique, especially for the studies of different petroleum samples (see Section 3.3 for details) [22]. The limitation of conventional GC-EI-MS analysis arises mainly when there is non-volatile and/or thermally labile component(s) in the mixture, thus impeding the compound coverage of GC-MS [12]. Advances in high-temperature GC-MS methods have enabled studies of compounds with boiling points up to 430 °C, which has largely expanded the compound coverage for GC-MS analysis [23]. Liquid chromatography can avoid this issue as its separation is not dependent on the boiling points of the analytes, it has been successfully coupled to various types of soft ionization sources and mass analyzers (see Section 2.3), and it has been applied in many disciplines for the purpose of structural elucidation [24,25,26]; however, modifications must be made when attempting to couple LC with EI-MS. EI is operated under high vacuum conditions, whereas the operating pressure of LC separation is usually considerably higher (i.e., the large amount of mobile phase used in LC separation must be removed before entering the EI source) [27]. Several adjustments have been made in order to overcome the aforementioned limitations, mainly via instrumental modifications to eliminate or reduce the amount of the mobile phase in the eluents before EI ionization. This was achieved by either adding a separate chamber in the LC-EI-MS interface for mobile phase evaporation [28] or applying a much smaller amount of the LC mobile phase (i.e., the application of the nanoLC technique or the supersonic molecular beam interface coupled with EI) [29,30]. Additionally, modifications of the EI source have also been achieved for the development of more powerful structural elucidation tools. For example, the utilization of cold EI allows the enhancement of the molecular ion’s (precursor ion) signal, thus enhancing the molecular characterization capability by providing the MW of the analytes [31]. The application of liquid EI systems has further expanded the MW range, and analytes with higher MW can be analyzed using LC-EI-MS [28]. Figure 1 shows several different setups of LC-EI-MS interfaces.

Another main advantage of LC-EI-MS techniques is that it is free from matrix effects; therefore, quantitative analysis is not affected by ion suppression as the ionization is solely dependent on the concentration of the analytes. Moreover, it is also free from the influence of salts and modifiers in LC eluents [27]. With all the modifications, LC-EI-MS has been used a great deal for the identification and quantification of different compound classes, including pesticides [32,33], drugs [29], and free fatty acids [30,34]. Normally, the most common mass analyzer used in LC EI-MS studies is ToF, although FTICR and ion traps are also used. The main limitation of LC-EI-MS is its relatively low sensitivity, which is at least one order of magnitude lower than other techniques. Memory effects, due to the accumulation of the neutral molecules in the instrument interface, caused by the LC solvent vaporization and non-homogenous ionization, is another major concern when using this technique [27]. A detailed description of the development and applications of LC-EI-MS techniques can be found elsewhere [27].

### 2.3. Soft Ionization and Tandem MS Based on Collisional Activation

Soft ionization techniques such as electrospray ionization (ESI), atmospheric pressure chemical ionization (APCI), atmospheric pressure photoionization (APPI), and matrix-assisted laser desorption/ionization (MALDI) are currently most popular for the analysis of biomolecules such as proteins, lipids, and metabolites. One of the major advantages of these ionization techniques is that they are operated under ambient (open air) conditions, and thus, they are more compatible with HPLC and UHPLC systems, without the need for sophisticated instrument modifications. In particular, ESI is superior for protein analysis because of the generation of multiple intact charged protein ions, thus resulting in wider compound coverage for proteins with large MW; this allows for the analysis of native proteins in the gas phase [35,36]. The major difference between soft ionization techniques and EI is that they gently ionize the neutral molecules without depositing excess amounts of energy; therefore, it is almost not possible to observe any fragment ions in the full mass spectrum. It is a beneficial feature for mixture analysis because there will be no, or few, fragment ions interfering with (de)protonated analytes in the mixture, thus leading to a much more efficient and accurate MW determination. On the other hand, in order to obtain a more detailed structural information of the analyte ions, additional steps must be taken (i.e., complementary ion fragmentation procedures must be introduced). Tandem MS (MS*^n^*) is the technique that is commonly used to isolate and fragment the ions of interest from the analyte mixture to gain detailed structural information.

Generally, MS*^n^* experiments can be performed inside trapping instruments (e.g., linear ion traps or LIT, orbitraps, Fourier transform ion cyclotron resonance, or FTICR) by selecting and trapping the ion of interest in a trapping device controlled by an electric or magnetic field, followed by the collisional activation of the ions using either adventitious collision targets, such as inert gas molecules (e.g., N_2_, He, or Ar), or a surface to induce energy transfer, followed by fragmentation [37]. The fragment ions can be further isolated and subjected to additional fragmentation reactions for more detailed structural information. The isolation and fragmentation occur in the same space (the ion trap) sequentially in time, which is referred to as “tandem in time” approach [38]. The other approach is called “tandem in space”, where the precursor ion is selected in one mass analyzer and transferred to a second mass analyzer for fragmentation reactions. This usually takes place in hybrid instruments, such as triple quadrupole (QqQ), quadrupole-time-of-flight (Q-ToF), and ToF-ToF, LIT-FTICR, and LIT-orbitrap [39,40,41]. Nowadays, many commercial instruments are capable of performing multistage MS*^n^* experiments, both in time and in space.

The choice of ion activation techniques often has distinct impacts on the fragmentation pathways of the ions [37]. The time frame of the activation methods ranges from 10^−15^ s to 10^2^ s, and the internal energy deposited to the ions also varies. Fast activation methods (e.g., surface-induced dissociation (SID, activation time 10^−14^–10^−10^ s) [42], electron capture dissociation (ECD, activation time ~10^−15^ s) [43] and electron transfer dissociation (ETD, activation time ~10^−15^ s) [44]) are activation methods that fragment precursor ions so quickly that no other chemistry-based (e.g., additional unimolecular reactions) interactions can occur. These activation methods have been widely used for structural analysis, especially analysis of large molecules such as proteins (see details in Section 3.1) [42,45].

“Beam type” activation methods usually occur within 10^−6^–10^−4^ s, where a beam of precursor ions is directed to a designated collision chamber, it collides with the collision gas (e.g., N_2_, He, Ar, etc.) and fragments. This is a slow and relatively low-energy (1–100 eV per charge) activation method [46]. The dissociation rate in this regime is comparable with the residence time of the ions in the collision chamber. A typical mass spectrometer for performing beam-type CID is QqQ, where the second quadrupole (q) is used as the collision cell. Collisional activation in Q-ToF and ToF-ToF instruments also belong to beam type methods. Recently, the so called “higher-energy collision-induced dissociation” (HCD) in high-resolution Orbitrap mass spectrometry has been more broadly used, and it is also considered a beam-type CID event (with higher collision energy applied). Typically, the activation energy deposited can be as high as several thousand eV per charge, and the resulting fragmentation spectra are largely dependent on the collision energy deposited, as well as the collision gas used. Gas-phase reactions can occur during the beam-type CID process as the product ions generated are not actively cooled by the collision gas.

Precursor ions can also undergo a “slow heating” process where thousands of collisions take place and small amount of internal energy is deposited to the precursor ions upon each collision; therefore, the ions’ activation, deactivation, and dissociation are happening in parallel during collisional activation, and various gas-phase unimolecular reactions can occur (such as rearrangements), as the activation time frame is generally longer than that of unimolecular reactions [47]. Note that in the beam-type CID event, multiple collisions may also take place because the length of the q collision chamber is sometimes larger than the mean free path of the precursor ions, but the number of collisions is substantially lower than in the slow heating process; however, the multiple collision events in beam-type CID are intrinsically different from those that occur during the slow heating process because the collision energies and activation times for these two methods are different. Ion trap CID in linear quadrupole ion traps and sustained off-resonance irradiation (SORI) in FTICR are both slow heating activation approaches. All the CID techniques mentioned have been extensively applied when characterizing molecular structures [48,49,50,51,52].

Photodissociation methods, including ultraviolet photodissociation (UVPD) and infrared multiphoton dissociation (IRMPD), have shown their unique characteristics for structural elucidation experiments, especially UVPD. Ions absorb high-energy UV photon(s) during UVPD, leading to the excitation of ions; thus, some unique high-energy dissociation pathways can be observed [53]. Single wavelength initiated UVPD (e.g., 157 nm, 193 nm, and 266 nm) has been utilized for the structural analysis of various biomolecules, including proteins, lipids, and carbohydrates [54,55,56,57,58].

Moreover, in-source CID (ISCID) is considered as an alternative technique for instruments without MS*^n^* capabilities. ISCID takes place in the medium-pressure region between the ion source and the high-vacuum mass analyzer (i.e., the ion optics region) [7]. By manipulating the magnitude of the accelerating voltage that was originally used for ion transfer, precursor ions gain extra kinetic energy and collide with the adventitious gas molecules to induce fragmentation. The energy deposited in the ISCID event usually ranges from several eV to hundreds of eV. This is a method that unselectively fragments all the ions generated from the ion source (all ions get accelerated in this region); thus, complex mixtures may not be analyzed due to the overlap of fragmented ions that originated from different precursor ions.

CID mass spectra of different standard compounds have also been collected into libraries so that they can be used as references for fragmentation pattern matching of unknown analytes, which is especially useful in untargeted analysis.

### 2.4. Ion–Molecule Reactions

As discussed in Section 2.3, MS*^n^* is a powerful tool for structural elucidation; however, sometimes MS*^n^*-based CID cannot provide adequate structural information, especially when attempting to identify and differentiate (positional) isomers. Many isomeric species yield identical MS^2^ and even MS^3^ spectra; for instance, isomeric deprotonated *N*- and *O*-linked glucuronides yield identical product ions as detected in a linear quadrupole ion trap, thus it is not possible to discriminate between them in mixtures [59]. Lipid isomers that differ by the positions of C=C bonds also cannot be differentiated by simple CID experiments [60]. Moreover, since these isomers are highly similar in structure, using HPLC to separate them is usually not very efficient, thus making the analysis of mixtures containing those molecules extremely challenging. In some cases, these isomers differ from each other by carrying different functional groups that are connected to the backbone of the molecules in different ways; therefore, they tend to exhibit different reactivities toward a certain reagent. Diagnostic gas-phase ion-molecule reactions have been discovered to aid the identification of different functional groups, and they have been successfully coupled to LC-MS. Further CID experiments on the product ions of the ion-molecule reactions have also been carried out for more detailed structural elucidation of various molecules [4,61,62]. A more detailed description of its applications will be introduced in Section 3.

Typically, an ion-molecule reaction is initiated by the “solvation” of the reactant ion in the gas phase; namely, the formation of a reaction complex between the ion and the molecule. This process determines the total energy that the complex can gain for further reactions. Normally, only reactions with transition state (TS) energy lower than the gained solvation energy can proceed forward to obtain a product, and the reaction rate is controlled entropically by the TS energy [4]. In principle, these gas-phase reactions must be exothermic, but some slightly endothermic reactions have been observed in some simple proton transfer reactions [8].

**Figure 2:**
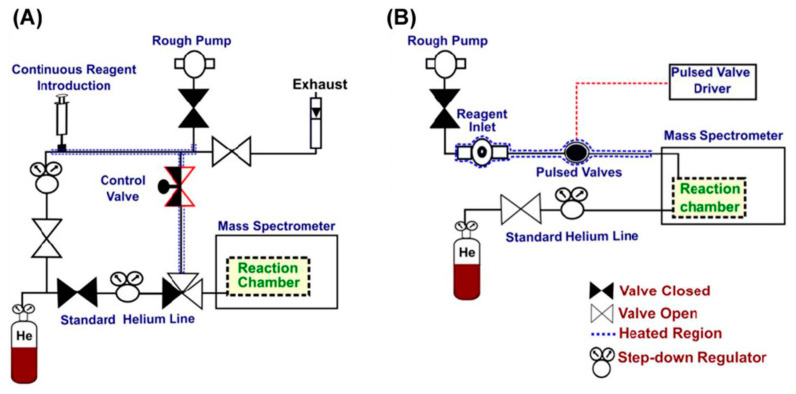
Reagent inlet systems for gas-phase ion-molecule reactions. (**A**) A reagent mixing manifold for continuous reaction kinetics monitoring, and (**B**) a pulsed valve system for fast reagent screening [4]. Adjusted and reprinted with permission from John Wiley and Sons.

A custom-built reagent inlet system is usually required for the introduction of the neutral reactants into the mass spectrometer. Several systems have been reported for efficient reagent introduction, either for screening fast multiple reagents or monitoring continuous reaction kinetics (Figure 2) [63,64,65]. Recently, a modified reagent mixing manifold was manufactured, and it can diminish the influence of adventitious molecules, such as water, on the reactions (a water-rich reaction chamber in the gas phase normally leads to dominant water addition reactions, thus hindering other diagnostic reactions), thus providing a constant and excess flow of neutral reactants for reactions [66]. Moreover, laser-induced acoustic desorption has also been used for the introduction of thermally labile solid reagents into the gas phase for ion-molecule reactions [67]. Mass spectrometers used for ion-molecule reactions are typically trapping instruments, such as LQIT, orbitraps and FTICR. Fusion instruments can also be used with proper modifications. With all the advances in instrumentation, and with more diagnostic neutral reagents and reactions identified, ion-molecule reactions have been, and will be, continuously used for structural elucidation purposes, especially in the analysis of complex biological samples.

### 2.5. Ion–Ion Reactions

In addition to gas-phase ion-molecule reactions, the interactions between two charged species are regarded as an alternative approach for providing structural information. The so-called ion–ion reactions usually involve an interaction between two oppositely charged ions. In order to observe the product ions in the mass spectrometer, the net charges that are usually carried by the two reacting species are different (e.g., a singly charged cation reacting with a doubly charged anion), leading to the formation of an adduct (or a Coulombic-bound complex) or an ion-transferred product. Single or multiple proton transfer, cation and/or anion transfer, and metal ion transfer, are typically observed during ion–ion reactions. Moreover, redox reactions may occur during the reaction, which is the result of electron transfer-based ion–ion reactions [68].

**Figure 3:**
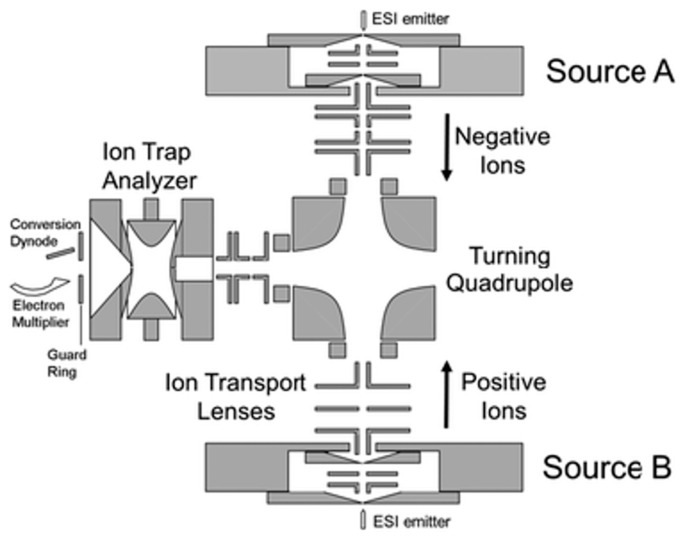
A sequential injection ion inlet system coupled with an ion trap mass spectrometer for gas-phase ion–ion reactions [69]. Adjusted and reprinted with permission from the Royal Society of Chemistry.

Ion–ion reactions are highly exothermic and efficient, as they are controlled by the Coulombic attractions of the two reactant ions. On the other hand, more sophisticated control of the reactant ions must be taken to ensure the high efficiency of the reactions, and instrument and software modifications must be made to ensure the proper overlap of the two reactant ions in space and in time [68]. The instrument used is usually equipped with two separate ion sources, so that ions with opposite charges can be generated without interference (Figure 3) [69]. The ions are then manipulated and ejected into the reaction chamber sequentially for further reactions. To diminish the influence of external electric fields on the reactions, ion–ion reactions are normally performed either under atmospheric pressure before entering the mass spectrometer, or in an electrodynamic ion trap, such as a linear ion trap or a 3D ion trap. Fusion instruments containing ion traps are also used [69]. Similarly to ion–molecule reactions, one of the reactant ions is in excess to achieve the maximal reaction efficiency.

Ion–ion reactions are a powerful means of analyzing large biomolecules, including peptides and proteins, thus providing simplified mass spectra by manipulating the charge states of the peptide and protein ions and yielding more detailed structural information (see examples in Section 3.1). In recent years, the applications of this technique keep expanding with the identification of an increasing number of selective and diagnostic ion-ion reactions, thus demonstrating its capability as another compelling structural elucidation tool in addition to ion–molecule reactions.

### 2.6. Ion Mobility Spectrometry-MS

**Figure 4:**
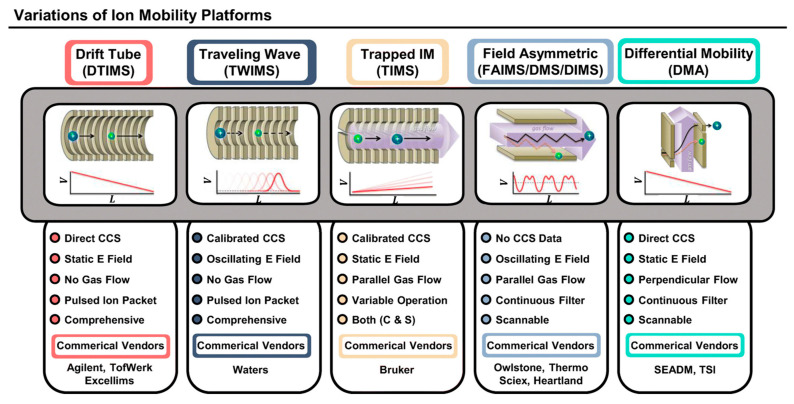
Schematic and features of different ion mobility platforms [18]. Adjusted and reprinted with permission from the American Chemical Society.

As previously mentioned, the separation of isomers using HPLC can be complicated because of the similarities between these molecules with regard to their affinity toward the stationary phase; therefore, additional separation techniques are in demand. Ion mobility spectrometry (IMS) separates ions based on their different shapes and sizes, which are reflected by their mobilities or collisional cross sections (CCS) [18]. IMS provides an additional dimension of separation so that isomers with different CCS can be separated, thus enabling increased peak capacity and high throughput analysis (Figure 4); therefore, various types of IMS instruments have been developed and coupled with HPLC and MS systems for structural elucidation purposes.

IMS-MS has shown its ability to separate and annotate/identify many isomeric biomolecules, including proteins, amino acids, lipids, and carbohydrates (see details in Section 3). A typical IMS-MS annotation workflow includes accurate mass measurements (and molecular formula prediction), isotope ratio calculations, MS/MS fragmentation patterns, and matching CCS values [18]; therefore, obtaining accurate CCS values of the analytes is crucial for IMS structural elucidation. CCS values can be directly calculated by measuring the mobility of ions using drift tube IMS. Other commonly used IMS instruments, such as traveling wave IMS and trapped IMS, require CCS calibration using ions with known CCS values for the calculation of CCS values of unknown analytes [70,71]. Field asymmetric waveform IMS cannot provide CCS values due to the application of an asymmetric waveform during mobility separation [72]. As with EI-MS, CCS libraries have been established for the purpose of identifying unknown analytes [73,74,75]. Moreover, machine learning/deep learning has been used to predict CCS values that are not available in the libraries, and the corresponding tools that were developed have successfully predicted CCS values for a broad range of analytes with reasonable median relative errors (in general, ≤3%) [76,77,78]. These tools have become increasingly important in multi-omics studies.

Although challenges still exist in the field of IMS-MS, especially for structural elucidation and omics studies, such as improving the IMS resolving power, obtaining CCS values with high confidence, and requiring widely accepted primary standards, this technique has proven to be significant, and it will keep expanding its applications in the near future [79].

### 2.7. Coupling MS with Other Analytical Tools

There are numerous structural elucidation tools that exhibit unique features other than MS, including nuclear magnetic resonance (NMR) [80], X-ray crystallography [81], and various other spectroscopic [82,83,84] and microscopic techniques [85,86]. These techniques are sometimes used together with MS in order to obtain more comprehensive structural information [87,88,89,90]. Computational approaches (for instance, computer assisted structure elucidation, or CASE [91]) are also frequently used to assist the structural prediction process [92].

Among all the aforementioned methods, NMR is the most popular technique that has been widely coupled with MS*^n^* and HPLC in a variety of studies [89,93]. A recent study demonstrated the abilities of NMR, and found that it is an important addition to MS as it can expand the coverage of metabolites in *Chlamydomonas reinhardtii*. In this work, in addition to the metabolites detected and identified by MS, another 20 metabolites were detected by NMR, and the structures of 14 of the detected metabolites were confirmed [93]. NMR exhibits unparalleled abilities to provide detailed and high throughput molecular profiling in biological samples, especially with the continuing advancement of two-dimensional (2D) NMR techniques (i.e., it can provide accurate atom connectivity information) [94]. Furthermore, the high reproducibility of NMR experiments makes it an even more powerful tool for structural elucidation studies. With the help of high-resolution (HR) MS and computational approaches, many unknowns can now be accurately predicted and identified in multiomics studies, thus allowing an enhanced understanding of the biological significance of these compounds during different biochemical processes [89,92]. Potential disease and injury biomarkers can also be identified with minimal bias so that early-stage disease diagnosis may become available [95,96]. Some recent studies that were using these techniques will be discussed in detail in Section 3.1, Section 3.2 and Section 3.3. Other than omics studies, NMR, (HR)MS, X-ray, and other spectroscopic methods (IR, UV-Vis, etc.) have been used as “standard” analytical tools for the structural elucidation of natural products and synthetic compounds [97] (see Section 3.5 for more details).

## 3. Applications of Mass Spectrometry-Based Techniques for Structural Analysis

In this section, we aim to focus on the applications of the MS-based structural elucidation techniques, and we provide a comprehensive overview of the technical advances that have been made in different fields since 2017, including various omics studies, studies of natural and synthetic compounds, and environmentally relevant compounds. Some special cases, such as the application of these analytical tools to capture and identify key reaction intermediates and the development of new computational approaches, will also be discussed.

### 3.1. Applications in Proteomics

As well as nucleic acids, proteins (and peptides) are the most important biopolymers that provide building blocks to cells and organisms, and that support their functionalities, transporting biomolecules to and from different cells, and acting as biocatalysts for many metabolic reactions [98]. The structures of proteins differ according to the species and number of amino acids in the peptide chain, the various folding patterns, and different post translational modifications (PTM). The interaction of proteins with other biomolecules, such as oligosaccharides, provides them with even more functionalities. The structures of proteins determine their different biological functions, and thus, it is important to elucidate their structures and understand their structural differences at the molecular level. Proteomics is the systematic analysis of proteins and proteomes on a large scale, and mass spectrometry has become the central technique for proteomic studies because of its incomparable sensitivity, throughput, and versatility.

MS-based protein structural elucidation can be categorized as “top-down” and “bottom-up” (shotgun) approaches. Top-down approaches study the intact protein ions in the gas phase using MS, and they perform follow-up dissociation experiments to obtain structural information. It is a reliable protein ID technique, but the throughput of the experiments is relatively low [99]. On the other hand, bottom-up or shotgun approaches study chemically or enzymatically digested proteins, and the proteolylic peptides generated are subject to HPLC-MS and MS/MS analysis and compared with libraries for structural elucidation. Although bottom-up approaches are highly sensitive and they deliver a high throughput, they suffer from limitations, such as the loss of key protein structural information [100].

#### 3.1.1. Studies of PTMs

Protein PTMs are mostly studied using bottom-up approaches. In these studies, loss of a (labile) PTM connection upon digestion has been an issue that needs to be addressed; therefore, efforts have been made to minimize PTM loss during analysis. First, different peptide enrichment methods have been developed so that some specific peptide species can be efficiently enriched. For example, a triarylphosphine–trimethylpiperidine reagent was used for the selective derivatization and enrichment of azide-labelled PTM peptides with enhanced ionization and fragmentation efficiency, and 3293 *O*-GlcNAc peptides have been identified in HeLa cells [101]. Many other labile and low-level PTMs have also been successfully enriched using different reagents and approaches. In a separate study, in order to enrich the extremely low-level nitrotyrosine peptides, a selective immunoaffinity enrichment method was applied, leading to almost 2000 identified nitrotyrosine sites from the whole-cell extract [102]. Overall, an increasing number of affinity-based and chemical-based enrichment methods have been recently developed for different target PTMs and amino acid residues [103].

Upon enrichment, MS/MS are normally performed for the structural elucidation of PTMs. CID, HCD, ExD (ECD or ETD), and UVPD are the most commonly used fragmentation techniques. Among them, CID is most commonly used. For example, a recent study used LC-MS/MS, based on CID, to identify enriched ADP-ribosylated proteomes, and it investigated their relevance in living cells, noting the effects of environmental changes and drugs [104]; however, sometimes CID tend to yield MS/MS spectra that are dominated by the fragment ion that has lost the original modifications, therefore, other collisional activation methods have been used either alone or in combination with CID (e.g., EthcD [105], EtciD [106], etc.) as alternative ways to study PTM structures. In particular, 193 nm UVPD is a powerful tool for cleaving peptide bonds in proteins [107], and the outstanding sequence coverage of UVPD makes it an increasingly popular method for protein PTM structural analysis, compared with the other fragmentation techniques. For instance, a comparative study using HCD, EThc, and UVPD on histone proteoforms showed that UVPD offered greater sequence coverage compared with HCD, especially for large proteoforms. A comprehensive proteoform identification was achieved by combining all three MS/MS techniques [108]. Hybrid fragmentation techniques, such as EThcD and ETciD, have been broadly used for structural characterizations of glycoproteins, one of the most common and important PTMs [50,105,106]. Structural elucidations of glycans will be discussed further in Section 3.3.

#### 3.1.2. Gas-Phase Ion Chemistry for Protein Structural Elucidation 

**Figure 5:**
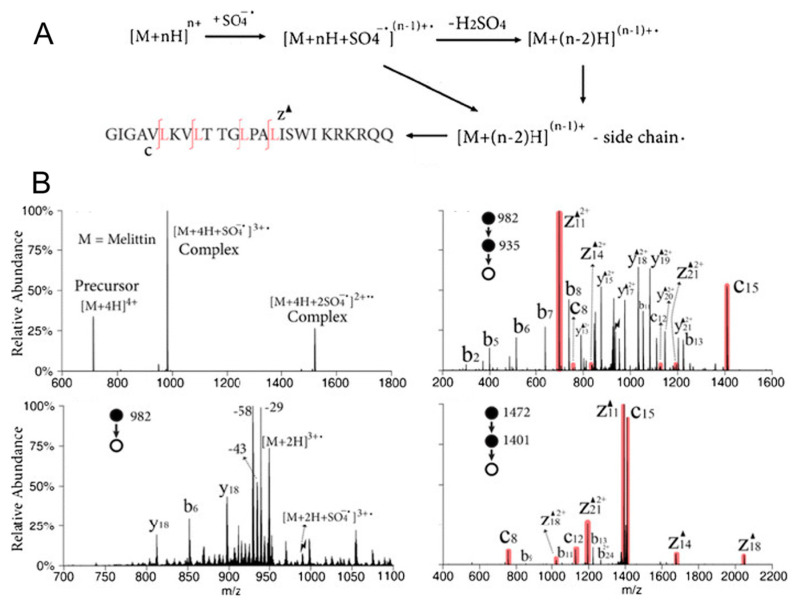
(**A**) Ion–ion reactions between sulfate radical anions and [M + 4H]^4+^ of melittin and (**B**) multi-stage CID mass spectra for the structural elucidation of the peptide [111]. Adjusted and reprinted with permission from the American Chemical Society.

As mentioned in Section 2, in addition to CID and other collisional activation methods, gas-phase ion–ion and ion–molecule reactions are also compelling approaches for structural elucidation; therefore, they have shown great potential in analyzing protein structures. Charge inversion ion–ion reactions, using a guanidinium-containing dicationic reagent, has successfully distinguished sulfopeptides from phosphopeptides, and the basis for such discrimination lies in the fact that there exists differential electrostatic interactions between the dication and the deprotonated peptide species [109]. In a separate study from the same research group, a synthetic 1-hydroxybenzoyl triazole (HOBt) ester (a singly charged cation) was used to react with dianionic peptides, followed by CID, to probe the zwitterion structures of the peptides in the gas phase [110]. Moreover, selective polypeptide cleavage has been achieved by using ion–ion reactions between sulfate radical anions and multiple protonated peptide ions in order to generate dehydroalanine residues in the peptide, thus providing additional potential methods for top-down protein structural characterization (Figure 5) [111].

Ion-molecule reactions have also been used for protein structural manipulations. Reactions between multiple charged protein ions can react with trimethylamine and result in a protein ion charge state concentration so that the protein ion signals are “merged” to a one charge state, which subsequently become more abundant [112]. We believe that with the discovery of an increasing number of ion–ion and ion–molecule reactions involving proteins and peptides, the gas-phase structures of such reactions can be more easily characterized and manipulated.

#### 3.1.3. Ion Mobility in Protein Structural Elucidation

Three-dimensional (3D) protein structures have also been well characterized by a MS-based technique called collision induced unfolding (CIU). Protein ions are activated to collide by some adventitious gas molecules, and they cause conformation changes in the gas phase [113]. Typically, the protein unfolding is directly characterized by the CCS change; therefore, IMS is usually used to directly observe and analyze the 3D protein structures. This technique has been applied to study membrane proteins, and different binding ligands to the proteins have been successfully classified based on different CIU signatures [114]. Moreover, the conformation of IgG4 monoclonal antibodies have been characterized by CIU IM-MS techniques, different conformational states and transitions have been observed by CIU experiments, and unfolding pathways of IgG4 species have been identified based on unique structural signatures revealed by CIU [115].

Tertiary protein structures have also been well characterized by IMS with the assistance of computational modeling. In a recent study, a Rosetta Projection Approximation with Rough Circular Shapes (PARCS) algorithm was developed to accurately predict the CCS of proteins, and thus provide evidence for protein structure prediction [116]. Moreover, stable structures of ubiquitin at different thermal denaturation conditions have been carefully studied and characterized by IM-MS techniques which couple with a temperature-controlled ESI source. Some well-defined structures are found to be stabilized as the temperature is increased [117].

There remain many more technical advances in MS-based methods, which are used for protein structural elucidation, that cannot be fully covered in this review, but we are confident that the aforementioned aspects that are used for protein analysis will keep their popularity, and expand and couple with new techniques for a more comprehensive understanding of protein structures and functions at the molecular level.

### 3.2. Applications in Lipidomics and Metabolomics

#### 3.2.1. Structural Elucidation of Lipids

As with proteins, lipids are another group of crucial biomolecules, with much lower MW, that are involved in various biological processes, including energy storage [118], building cell membranes [119], participating in immune response [120], and cell signaling [121]. They are also excellent biomarkers that can be potentially used for the diagnosis of diseases such as cancers in the early stages [122]; however, the complexity of lipids that are reflected by different hydrophilic head groups, alkyl tail chain lengths, C=C bond positions in the alkyl chains, and the relative positions of the alkyl chains (i.e., *sn*-positions), makes the analysis of lipids extremely hard, especially in complex biological mixtures. In order to obtain unambiguous structural information regarding lipids, an increasing number of MS-based analytical tools have been recently established.

Normally, lipid head group information can be obtained using MS/MS experiments, as most of the lipids will yield diagnostic fragment ions that can be used as signatures of their head groups [123]. Obtaining further structural information concerning lipids, beyond the head group identity, simply by using MS/MS techniques is challenging. Efforts such as chemical derivatization, gas-phase ion–molecule and ion–ion reactions, photochemistry, and unique fragmentation approaches have been made to enhance the ability of MS-based lipid structural elucidation techniques.

**Figure 6:**
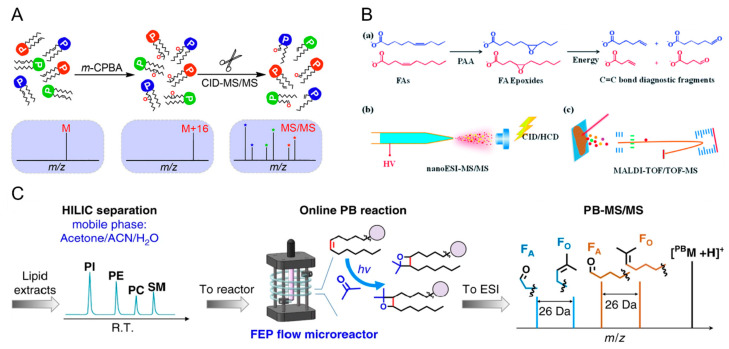
Lipid C=C bond localizations using (**A**) *m*-CPBA epoxidation [124], (**B**) PAA epoxidation [125], and (**C**) an online P-B reaction [126], followed by MS/MS for the structural elucidation of lipid isomers. FA, fatty acid, PI, phosphatidylinositol, PE, phosphotidylethanolamine, PC, phosphotidylcholine, SM, sphingomyelin, ACN, acetonitrile. Adjusted and reprinted with permissions from the American Chemical Society (**A**), the Royal Society of Chemistry (**B**), and Springer Nature (**C**).

In order to pinpoint the C=C bond positions, and differentiate these double bond positional lipid isomers, epoxidations of C=C bonds using *meta*-chloroperoxybenzoic acid (*m*-CPBA) have been conducted in solution. Upon the ionization and CID of the epoxidized lipids, signature fragment ions containing terminal carbonyl groups in the lipid alkyl chains are detected, thus enabling the large-scale characterization of lipid isomers (Figure 6A) [124]. Similarly, this method was further modified and adapted to the mass spectrometry imaging (MSI) studies of fatty acids. By performing on-tissue derivatization using peracetic acid (PAA), the epoxidation of C=C bonds in unsaturated fatty acids enables the generation of diagnostic fragment ions upon MS/MS. It provides a simple and efficient approach for fatty acid identification in MSI experiments (Figure 6B) [125]. The Paternò–Büchi (P-B) reaction is another online photochemical derivatization method that has been widely used to differentiate lipid isomers. Recently, a large-scale MS-based analysis of phospholipid isomers was carried out, and the C=C positions of more than 200 unsaturated glycerophospholipids in bovine liver were localized (Figure 6C) [126].

Several gas-phase ion–ion reactions have also been developed and used for lipid C=C localization. For example, C=C bond positions and cyclopropane sites in cardiolipins have recently been determined by using charge inversion reactions of deprotonated cardiolipins and tris-phenanthroline magnesium reagent dications. Several isomeric cardiolipins that make different contributions to the carbocycles of *E. coli* have been identified [127]. In a separate study, structures of ether glycerophospholipids were elucidated by using charge inversion ion–ion reactions for [MgPhen_3_]^2+^ ions [128]. Furthermore, such charge inversion ion–ion reactions have been successfully coupled with MSI experiments by combining the reactions with 1,4-phenylenedipropionic acid dianions. Isomeric phosphatidylcholine (PC) lipid cations have been identified upon CID in MSI experiments using rat brain tissues [129].

Lipid structures can also be characterized by using simple dissociation methods. Unlike conventional collisional activation methods that can only provide lipid head group information, UVPD has successfully proven that it can selectively cleave C-C bonds that are adjacent to the C=C bonds in the lipid alkyl chains. This leads to the generation of a group of diagnostic fragment ions with a mass difference of 24 Da. Isomeric phosphotidylcholines have been successfully identified in an orbitrap mass spectrometer by using both 193 nm UVPD and HCD [130]. A similar approach combining 193 nm UVPD and CID was applied in a different study to localize the C=C bonds in cardiolipins. C=C bonds in the four alkyl chains of cardiolipins were accurately pinpointed [131].

Ozone-induced dissociation (OzID) is another reaction-based dissociation method that is widely used for lipid C=C bond pinpointing. Ozone is generated and introduced to the mass spectrometer, and C=C bonds are selectively cleaved in the lipid alkyl chains to yield signature fragment ions. Recently, this technique has been coupled with different instruments, such as IM-MS instruments, to achieve the efficient separation and identification of lipids in biological samples [132,133,134]. High-pressure OzID performed in the IMS cell has shown that this technique can be used on a transmission timescale, thus suggesting its potential applications in data-dependent and independent lipidomic experiments [135]. Moreover, OzID applied in MSI studies enables the unambiguous visualization and localization of isomeric lipids in different tissue sections, thus revealing that selective lipid synthesis is closely related to the types of tissue subsections and their environment [136].

#### 3.2.2. Structural Elucidation of Metabolites

Small molecule metabolites can also be identified via MS-based techniques. HPLC-MS/MS is a typical workflow for metabolite ID, and several metabolite databases have been established to aid with efficient and accurate identification [137,138]. Recently, a structural similarity scoring system based on MS/MS data was developed and applied to identify unknown metabolites. The unknown metabolites are scored and compared against structurally related known metabolites in the databases, in order to largely expand the metabolite identification coverage [139].

**Figure 7:**
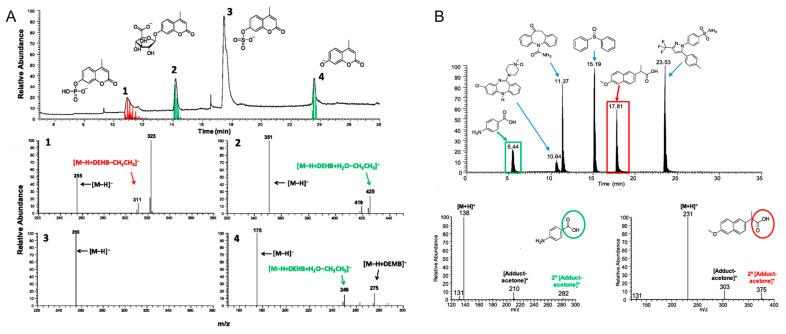
(**A**) Identification and differentiation of a deprotonated hypercromone and its three metabolites using HPLC coupled with diagnostic ion–molecule reactions using diethylmethoxyborane and water [140]. Diagnostic products are labelled in red and green. DEHB and diethylhydroxyborane are produced upon hydrolysis of DEMB. (**B**) The identification of carboxylic acids in a mixture containing compounds, along with other functional groups, using HPLC coupled with diagnostic ion–molecule reactions with (isopropenoxy)trimethylsilane [141]. Diagnostic products are labelled in red and green. Adjusted and reprinted with permissions from the American Chemical Society (**A**) and Elsevier (**B**).

The ion–molecule reaction is another powerful tool for metabolite identification. As previously mentioned, various diagnostic ion–molecule reactions have been identified so that isomeric compounds with different functional groups can be identified and differentiated. These reactions have been used for the differentiation of different metabolites; for instance, a deprotonated hypercromone and its three metabolites (a phosphate, a sulfate, and a glucuronide) has been successfully differentiated using an ion–molecule reaction-based HPLC-MS/MS workflow (ion–molecule reactions with diethylmethoxyborane and water), thus demonstrating that ion–molecule reactions are highly efficient approaches that can be incorporated well with HPLC separations (Figure 7A) [140]. Drug metabolite model compounds that contain carboxylic acid functionalities have also been identified and differentiated from other metabolites including sulfones, hydroxylamines, sulfonamides, ketones, amines, N-oxides, sulfoxides, and carboxamides using diagnostic ion-molecule reactions with (isopropenoxy)trimethylsilane in positive ion mode (Figure 7B) [141]. Moreover, a machine learning algorithm has been established to predict ion–molecule reaction products, and to rule out certain functional groups that cannot show specific diagnostic products upon reacting with the selected neutral reagent [142].

An approach combining NMR and MS/MS has been developed for the identification of secondary metabolites in *Arabidopsis thaliana*. Unknown metabolites in different systems including plants, microbes, and biomedical samples can be identified via this hybrid technique, and the metabolomics database can be further expanded [89].

With the requirement for the more accurate and efficient lipid and metabolite identification, we believe that more methods will be developed based on ultra-high resolution mass measurement and more powerful separation techniques. Data processing techniques also need to be developed so that large-scale data generated in experiments, such as MSI, can be analyzed more efficiently. Machine learning will continue to develop in this field to help elucidate lipid and metabolite structures with higher confidence 

### 3.3. Applications in Glycomics

Glycans are key biomolecules that are involved in many biological processes, such as cell–cell interactions, cell adhesion, signaling, and proliferation [143,144]. Normally glycans are connected with other biomolecules such as proteins, peptides, and lipids via glycosylation. Two types of glycans exist in glycoproteins and glycopeptides (i.e., *N*-glycans and *O*-glycans) as a result of bonding with the different amino acid residues of proteins. Glycolipids are formed by bonding carbohydrate molecules to the lipid via glycosidic bonds. Uncovering glycan and glycolipid structures, and studying their relevant biofunctionalities, is challenging due to the diversity of these molecules in terms of their different linkages and sequencings. MS-based methods are becoming the core technologies with which to reveal glycan structures for disease pathogenesis studies and biomarker discoveries [145].

Similarly to proteomic and lipidomic studies, HPLC-MS/MS is commonly used in glycomic studies to elucidate different glycan structures. A technique called logically derived sequence tandem mass spectrometry has been used to automatically determine different glycan structures. Sequential CID experiments on different oligosaccharides were carefully selected and performed under the guidance of a logically derived sequence, and the complete structural information was obtained from the selected CID events. Several oligosaccharides and *N*-glycan isomers have been identified using this approach in separate studies [146,147]. Similarly, an intelligent precursor selection method has been developed for the selection of proper precursor ions for sequential glycan MS*^n^* experiments. The precursor ions were selected based on the probability (and “power”) of them providing adequate glycan structural information in the subsequent CID steps. Fifteen glycan standards were distinguished based on this method [148]. In the HPLC separation of glycans, instead of commonly used reverse phase columns, a nanoflow with porous graphitized carbon chromatography was coupled with MS/MS to characterize the native sulfoglycans in different vaccines. The potential of replacing sialyation using glycan sulfation in vaccine design processes has been evaluated [149].

Other than HPLC, different separation techniques have also been used for glycan structural elucidation; for example, capillary (microchip) electrophoresis (CE) has been used with orbitrap MS for direct *N*-glycan identifications in human serum. In this study, CE provided high resolution separations of *N*-glycans, 77 of them have been detected and unambiguously identified in the follow up CID experiments, and 31 of the detected *N*-glycans were new species [150].

**Figure 8:**
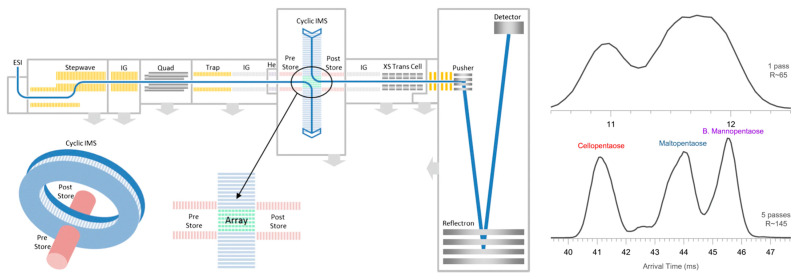
The geometry of the cIM-ToF mass spectrometer (left) and the increase in mobility resolving power by using 5-pass mobility separation in the cIM chamber for differentiating between different pentasaccharides [153]. Adjusted and reprinted with permission from Springer.

Ion mobility has improved the structural analysis of glycans to a large extent. By matching the CCS values and the fragmentation patterns of the glycan ions, an increasing number of unknown glycans has been identified using IM-MS techniques. An ion mobility-based separation method has been applied in both positive and negative ion mode, to identify sodiated ([M + Na]^+^) and deprotonated ([M − H]^–^) oligosaccharides using both mobility and *m/z* profiling. This study also demonstrated how the addition of divalent metal ions, or the attachment of two monovalent metal ions, can further enhance the mobility separations of glycan isomers [151]. A gate-trapped ion mobility instrument was coupled to a FTICR mass spectrometer that uses electronic excitation dissociation (EED) for MS/MS experiments in order to elucidate isomeric glycan structures. This method provides an enhanced mobility separation, and EED spectra show extensive fragment ions that are independent of the gas-phase conformations of the glycans, thus enabling much better isomer differentiation capabilities [152]. A cyclic IM (cIM) chamber was built and coupled with a Q-ToF mass spectrometer for distinguishing different pentasaccharide anomers and open-ring forms. The cIM chamber allows mobility separations of the analytes by performing multi-pass separation experiments (i.e., ions are cycling within the IM chamber for multiple cycles), thus leading to a higher resolving power in the arrival time distributions of pentasaccharides (Figure 8) [153].

As mentioned in Section 3.1, hybrid fragmentation techniques have also been extensively used for glycoprotein and glycopeptide structural analysis.

### 3.4. Applications in Petroleomics

Petroleum is another example of a highly diverse and complicated mixture that consists of hydrocarbons and hetero atom-containing hydrocarbons. Analysis of petroleum samples by MS can be challenging because of its structural complexity, extensive fragmentation upon hard ionization, such as EI (fragment ions overlap with other components in the full mass spectrum, and the MW of hydrocarbon molecules are not available), and poor ionization efficiency using soft ionization techniques such as APCI and APPI [154]. Instead of performing conventional GC-MS experiments, two-dimensional GC (2D GC or GC × GC) EI-MS have been extensively used for the analysis of petroleum samples, thus leading to greater separation, sensitivity, and compound coverage [22]. To overcome the limitations of EI, namely the extensive fragmentation that can occur during ionization, several ionization techniques have been used together to retain the MW information and increase the compound coverage. For example, a base oil sample has been separated using GC × GC, followed by ionization and analysis using EI-, PI-, CI- and FI-ToF-MS (FI: field ionization). Among these ionization techniques, FI generates the most intact molecular ions, regardless of hydrocarbon classes, CI tends to produce protonated molecules and pseudo molecular ions upon hydride abstraction for certain hydrocarbon classes, including steranes, thiophenes, and esters. For branched alkanes and saturated monocyclic hydrocarbons, PI can retain the MW information and generate specific fragment ions at the same time. EI, however, tends to extensively fragment other hydrocarbon classes, and is well suited for retaining MW and providing unique fragmentation patterns for bicyclic/polycyclic naphthenic and aromatic compounds; therefore, different hydrocarbon molecules in the base oil have been systematically studied, thus leading to comprehensive hydrocarbon class coverage [154].

In addition to conventional ToF MS analysis, a GC-atmospheric pressure laser ionization (APLI)-TIMS-ToF-MS workflow has enabled the identification of polycyclic aromatic hydrocarbons (PAH) and other structurally similar compounds [155]. CCS values of the tested PAHs were reported for the first time, and they were used to assign potential candidate structures to EI-MS fragmentation patterns. Moreover, the use of GC-APLI can preserve MW information of the PAHs, thus enabling accurate mass measurement for molecular formula confirmation [155].

In order to improve the ionization efficiency of soft ionization techniques for the analysis of hydrocarbons, several modifications have been made. An APCI-MS method that uses oxygen as the reagent gas (conventional APCI uses nitrogen as the reagent gas), and *n*-hexane as the APCI reagent (for hydride abstraction from the hydrocarbon molecules), was used to study the hydrocarbon class distributions in a lubricant base oil sample. The results were compared with conventional FI-MS methods, and the APCI-O_2_-hexane approach showed that predominant pseudo molecular ions in the full mass spectrum with minimal fragmentation levels are comparable with FI-MS results. The average MW determined by these two methods are also similar; however, the reproducibility of the FI-MS experiments was found to be much worse than the APCI-O_2_-hexane approach [156].

**Figure 9:**
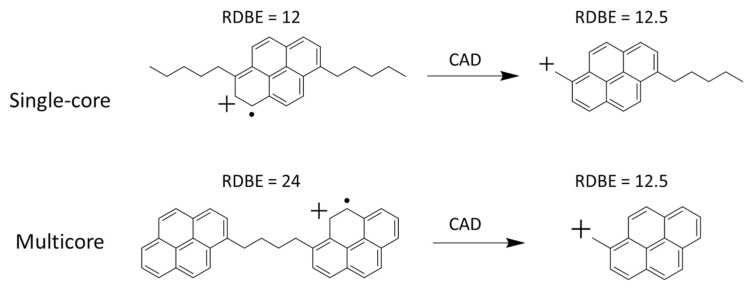
Fragmentation of a single core and multicore asphaltene molecular ions upon single stage CID [157]. The product ion generated upon CID of the single core precursor ion showed an increase in RDBE by 0.5, and the product ion generated upon CID of the multicore precursor ion showed a decrease in RDBE by 11.5. CAD, collision-activated dissociation, is the same technique as CID. Adjusted and reprinted with permission from Elsevier.

Asphaltenes are aromatic compounds existing in crude oil that cause serious problems in petroleum industry, such as clogging pipelines and fouling catalysts. Understanding the structures of asphaltenes can assist in resolving these issues. Asphaltenes possess two basic structural models, island and archipelago. The island model, or the single core model, refers to a single or fused aromatic ring connecting with several alkyl side chains. In an archipelago (multicore) model, several aromatic cores are connected with each other via alkyl chains. It is essential to learn the distribution of these two types of asphaltenes in crude oil samples so that action can be taken to reduce or avoid the side effects brought about by them. A method combining in-source CID and HCD (or medium-energy CID) in an orbitrap mass spectrometer has been established for the structural differentiation of the island vs. archipelago asphaltenes. Several asphaltene model compounds, and two crude oil samples, were examined. HCD and in-source CID results, respectively, showed that the changes in the ring and double bond equivalence were different for these two models upon fragmentation, thus suggesting a different ratio between these two types of asphaltenes in the crude oil samples, whereas conventional MS techniques tend to introduce sampling bias. The relative abundances of these two types of asphaltenes were also determined, providing guidance for oil refining processes (Figure 9) [157]. A detailed summary of the studies of asphaltene samples using different MS*^n^* techniques is described elsewhere [158].

High-resolution MS has been more extensively used in petroleomics studies for more accurate mass measurements. A high-field MegaOrbitrap Fourier transform mass spectrometer, equipped with ESI, has been used and evaluated for the characterization of crude oil samples. This high-resolution instrument is able to provide >1,000,000 mass resolving power at *m/z* 200, leading to sub-ppm mass accuracy. The detection time required to achieve such resolving power is ~3 s, which is well suited for analysis of complex crude oil mixtures [159]. In a separate study, an ESI-FTICR mass spectrometer and an ESI-orbitrap mass spectrometer were used for the analysis of four crude oil samples. These high-resolution techniques have been proven to provide a more sensitive and accurate assignment of hydrocarbon classes. Moreover, the more accurate DBE intensity plots generated by these methods allows for greater statistical certainty [160]. More information on the application of high-resolution MS in petroleomics studies can be found elsewhere [161].

### 3.5. Applications in Natural Products Structural Analysis

MS has been used for the structural elucidation of natural products for decades [162]. As previously mentioned in this paper, MS is a powerful and versatile tool that can provide important structural information, such as MW, molecular backbone, and functional group connectivity. It can also reveal the sequence and ratio of monomeric units in polymers (e.g., protein and peptide sequencing, see Section 3.1). In addition to the aforementioned biomolecules, many other naturally occurring compounds have been extensively characterized by MS and relevant methods. MS has several applications which can be used for the analysis of these compounds, and they are demonstrated in this section.

**Figure 10:**
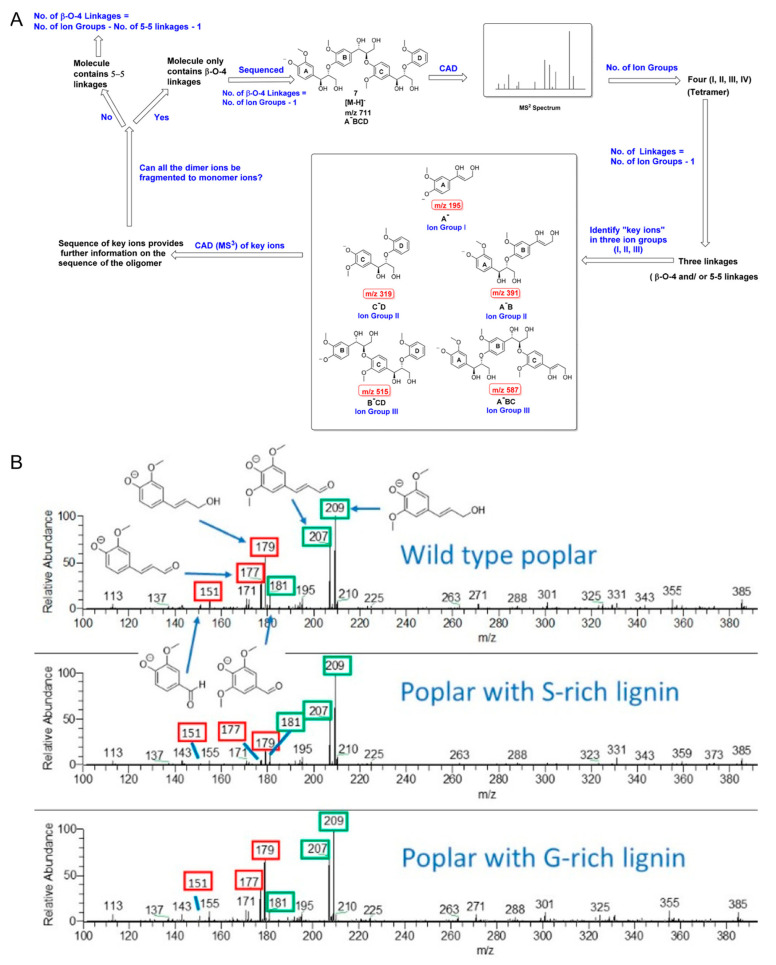
(**A**) A workflow for sequencing lignin oligomers with different linkages using multi-stage CID; key ion groups are shown in the black box [163]. (**B**) CID high-resolution mass spectra for three types of natural lignin samples. Fragment ions related to G-lignin are labelled in red boxes, and fragment ions related to S-lignin are labelled in green boxes. The ratio of the peak abundances of these fragment ions are used for the determination of G- and S-lignin content in the wild-type lignin samples [165]. Adjusted and reprinted with permissions from the American Chemical Society.

Lignin is the second most abundant biopolymer in plants and the most abundant aromatic species. Lignin has great potential as a feedstock for the sustainable generation of high-value aromatic compounds that can substitute for the excess use of crude oil [163] therefore, it is essential to understand lignin structures so that proper degradation methods can be applied accordingly to produce high-value small molecules. Lignin comprises a group of structurally complex chemicals that are constructed from three different monomeric units (G, S and H) [164]. These lignin monomers are connected with each other via many different linkage patterns, thus ensuring that the structural elucidation of lignin is not an easy task. MS*^n^* has been proven to be highly efficient for lignin sequencing. A large-scale lignin sequencing study has been conducted by multi-stage CID experiments on 10 different G- and S-lignin oligomers. Key fragment ion groups from MS^2^ and MS^3^ spectra are identified, providing important monomer connectivity information for lignin polymers (Figure 10A) [163]. Furthermore, the monomer compositions of lignin in several genetic variants of poplar has been determined using fast pyrolysis coupled with high resolution APCI-MS. This method was evaluated by comparing conventional derivatization methods, and it proved to be a simpler, faster, and more precise alternative approach (Figure 10B) [165].

Alkaloids are a group of nitrogen-containing basic natural products that have been widely used as medicines or substrates for new drugs [166]. For example, aporphine alkaloids are a group of isoquinoline-based alkaloids, and many of them show potential bioactivities, such as anti-diabetic, anti-oxidant, and anti-HIV activities [167]. A recent study using ESI-MS/MS and DFT calculations demonstrated the localization of the methylenedioxy, aromatic methoxy, vicinal methoxy, and hydroxy groups in 10 different 7,7-dimethylaporphine alkaloids, thus proving the ability of this method to quickly structurally characterize aporphine alkaloids [168].

Flavonoids comprise a group of major plant metabolites, which has shown high medicinal values and antioxidant activities [169]. Flavonoid metabolites in citrus peels have been characterized by a UPLC-MS/MS method, wherein 252 metabolites were identified, and several of the unique molecules were used to distinguish different citrus varieties [169]. In a different study, flavonoids from switchgrass and *Mikania micrantha* were detected and identified using an efficient HPLC-MS/MS method, three characteristic fragment ions were used to categorize different flavonoid types, and 10 unreported flavonoids were discovered in the studied plants, further demonstrating that MS is a powerful analytical tool for the structural characterization of natural products [170].

## 4. Conclusions and Future Perspectives

Mass spectrometry-based structural analysis techniques have emerged as keys to unlock the molecular structures of analytes, which are of critical importance in many fields, such as biochemistry and material science. Furthermore, these techniques have shown great potential in uncovering the relationship between molecular structures, their unique functions, and chemical and physical properties. We have demonstrated the capabilities and broad applications of MS to analyze structures of important biomolecules, hydrocarbon molecules, and natural products. Although sample preparation techniques are not discussed in this context, they are critical steps that need to be taken prior to MS analysis in order to ensure highly sensitive MS measurements, and to obtain reproducible results. Optimized sample preparation steps should be carefully undertaken to avoid possible variances or biases. A standard sample preparation protocol is desirable in many studies, especially for complex mixture analysis, such as multiomics studies [171,172]. For MS-based structural analysis, CID, and many other fragmentation techniques, have been extensively applied to the structural analysis. Implementing gas-phase reactions to these fragmentation techniques has further enhanced the power of such analyses, especially for the analysis of isomeric molecules. We believe that an increasing number of diagnostic reactions and reagents will be identified and used in the structural elucidation of different compound classes. More efficient diagnostic reactions, and more robust workflows for the high throughput analysis of more complex analytes with multiple functional groups, are still in demand. With the rapid development and expansion of ion mobility techniques, IM-MS methods have been increasingly applied to structural elucidation studies. We foresee the improvement of IM instrumentations, such as a higher mobility resolving power, which will facilitate the application of IM-MS into this field; coupling this technique with more analytical tools is also of potential interest. Furthermore, an increasing number of computational methods has been used with MS-based techniques to facilitate more efficient data processing and interpretation. As the analytical power of MS-based techniques increases (e.g., more compound coverage and higher mass resolving power), larger datasets will be generated; therefore, we are confident that machine learning and/or deep learning and other computational approaches will be increasingly used to assist data compression and filtering. Additionally, predictive models will be developed for future studies of unknown samples. In recent years, another rapidly growing application of MS is its use in the study of spatially resolved omics using mass spectrometry imaging (MSI) techniques. This approach allows direct, label-free visualization and spatial analysis (distributions and alterations of certain molecules of interests) using tissue sections collected from animals or plants. One of the major challenges of such a study concerns accurate molecular annotations/identifications. Increased efforts have been made to couple MS-based structural elucidation techniques with MSI experiments (see examples in Section 3), and this field will remain active [173]. Finally, the structural analysis of molecules in a confined environment, such as in single cells, will gain increasing popularity with the further improvement of MS techniques.

## Data Availability

Not applicable.
